# The Role of Surface Electromyography in Data Fusion with Inertial Sensors to Enhance Locomotion Recognition and Prediction

**DOI:** 10.3390/s21186291

**Published:** 2021-09-19

**Authors:** Lin Meng, Jun Pang, Ziyao Wang, Rui Xu, Dong Ming

**Affiliations:** 1 Academy of Medical Engineering and Translational Medicine, Tianjin University, Tianjin 300072, China; pangjun@tju.edu.cn; 2 International Engineering Institute, Tianjin University, Tianjin 300072, China; wangziyao@tju.edu.cn; 3 Department of Biomedical Engineering, School of Precision Instrument and Opto-Electronics Engineering, Tianjin University, Tianjin 300072, China; xrblue@tju.edu.cn

**Keywords:** data fusion, multimodal sensing, inertial sensor, surface electromyography, locomotion recognition, locomotion prediction, machine learning

## Abstract

Locomotion recognition and prediction is essential for real-time human–machine interactive control. The integration of electromyography (EMG) with mechanical sensors could improve the performance of locomotion recognition. However, the potential of EMG in motion prediction is rarely discussed. This paper firstly investigated the effect of surface EMG on the prediction of locomotion while integrated with inertial data. We collected EMG signals of lower limb muscle groups and linear acceleration data of lower limb segments from ten healthy participants in seven locomotion activities. Classification models were built based on four machine learning methods—support vector machine (SVM), k-nearest neighbor (KNN), artificial neural network (ANN), and linear discriminant analysis (LDA)—where a major vote strategy and a content constraint rule were utilized for improving the online performance of the classification decision. We compared four classifiers and further investigated the effect of data fusion on the online locomotion classification. The results showed that the SVM model with a sliding window size of 80 ms achieved the best recognition performance. The fusion of EMG signals does not only improve the recognition accuracy of steady-state locomotion activity from 90% (using acceleration data only) to 98% (using data fusion) but also enables the prediction of the next steady locomotion (∼370 ms). The study demonstrates that the employment of EMG in locomotion recognition could enhance online prediction performance.

## 1. Introduction

Movement disorders often occur as a manifestation of neurological diseases such as strokes, spinal cord injuries, and Parkinson’s disease [1]. Lower limb exoskeletons can augment the motor function of patients, enhance their locomotion abilities, and improve motion rehabilitation outcomes [2,3]. To restore the user’s normal natural gait patterns, an advanced human–machine interaction is essential to recognize and predict the locomotion states and dynamically adapt to different locomotion modes.

Various types of sensors have been utilized in the application of locomotion recognition. Among them, inertial measurement units (IMUs) that consist of accelerometers and gyroscopes have been widely employed in wearable systems due to their small size, low cost, and ease of use [4,5,6]. San-Segundo et al. [6] proposed a human activity recognition method based on a hidden Markov model that recognized six different daily activities: walking, walking upstairs, walking downstairs, sitting, standing, and lying. Haoyu Li et al. [4] developed an adaptive online classification model using a single IMU sensor placed on a shoe. The algorithm, based on a nonparametric triplet Markov model, obtained an overall accuracy above 98% for activity recognition of walking, running, stair ascent, and descent. Martinez-Hernandez and Dehghani-Sanij [5] introduced an adaptive Bayesian inference system method in which three sensors were used to recognize walking on different terrains such as level ground walking, a ramping ascent, and a descent. The Bayesian-based algorithm achieved a response time of 40 ms with an average accuracy of 99.82%. Studies have shown that the systems using inertial sensors can obtain a high classification accuracy in the recognition of activities. However, as inertial signals represent the current motion state of the human body segment, it is still challenging to predict the motion intention of locomotion activities using only inertial data.

As surface electromyography (sEMG) could directly represent muscle activation patterns reflecting the volitional control of human motion [7], it has been used in multiple studies to identify locomotion activities [8,9,10,11,12]. Huang et al. [13] developed a gait-phase-dependent model using sixteen channels of lower limb sEMG signals to identify seven locomotion modes. Xi et al. [8] investigated a series of sEMG features and recognition methods for daily activity monitoring using EMG signals of the rectus femoris, semitendinosus, tibialis anterior, and gastrocnemius. However, current EMG-based models cannot achieve a precise recognition performance compared with methods using inertial data due to a low signal-to-noise ratio and a high variability in EMG [7].

A few studies have considered fusing inertial data and sEMG for locomotion prediction, which was established by classifying transitions before the critical events of gait such as a heel strike or toe off. Peng et al. [14] proposed a multilevel classifier model in which sEMG was used to build a gait transition classification model and the fusion of sEMG and inertial sensors was used to build a steady-state locomotion recognition model. Hu et al. [15] proposed a double-model method with the integration of data from IMU and EMG sensors, which could accurately classify locomotion activities and gait phases. The transition between the locomotion activities and the steady-state modes were usually considered as independent tasks in the classification [16], which may result in the expense of using complicated classifiers and a computational burden for embedded robotic systems. However, the physiological property of sEMG that activates before an actual kinematic performance and its impact on the prediction of motion intention have been rarely discussed in current studies. We hypothesized that the employment of EMG enables the model built based on steady-state locomotion data to predict the next locomotion activity in advance during the gait transition (before the steady-state locomotion activity began).

In this paper, we investigated the impact of EMG-fused inertial data on locomotion recognition and prediction. Classification models were built based on four machine learning methods—support vector machine (SVM), k-nearest neighbor (KNN), artificial neural network (ANN), and linear discriminant analysis (LDA)—where a major vote strategy and a content constraint rule were utilized for the classification decision. Assuming that the methods should be able to predict steady locomotion activities during gait transitions, the effects of window sizes and the used data type for the online recognition performance based on the four different machine learning models were analyzed. Our results demonstrate that the classification models trained based on the data during steady-state locomotion can predict the locomotion modes during the gait transition. SEMG plays an essential role in enhancing the prediction ability of the classifiers whereas the prediction performance for different locomotions is varied. 

## 2. Methods

### 2.1. Experiment Setup

The study was approved by the Ethics Committee of Tianjin University and was conducted at the Motion Rehabilitation Lab, Tianjin University. Ten healthy young people (5 males and 5 females, age 23.00 ± 0.82 years old, weight 60.71 ± 11.25 kg, height 168.00 ± 9.40 cm) were enrolled in the experiment. The participants did not have any movement disorders and related neurological diseases. All participants provided written informed consent before the experiment.

**Figure 1:**
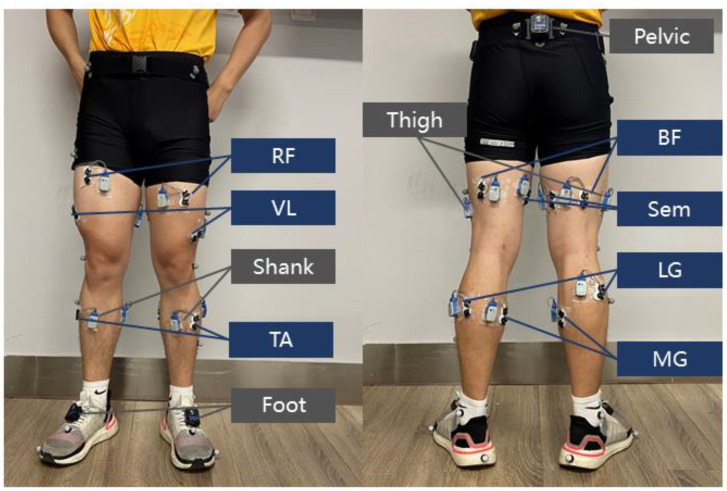
Experiment setup for data collection. The participant wore a set of sensors and retroreflective markers. EMG electrodes were placed on 14 muscles—rectus femoris (RF), vastus lateralis (VL), biceps femoris (BF), semitendinosus (Sem), tibialis anterior (TA), medial gastrocnemius (MG), and lateral gastrocnemius (LG)—of both legs. Motion sensors were placed on the pelvis, thigh, shank, and foot. A Plug-in-Gait (PiG) marker set was used as a reference for identifying the locomotion activities and gait cycles.

As shown in Figure 1, fourteen major muscles of both legs were selected, namely: rectus femoris (RF), vastus lateralis (VL), biceps femoris (BF), semitendinosus (Sem), tibialis anterior (TA), medial gastrocnemius (MG), and lateral gastrocnemius (LG). The linear acceleration of lower limb segments, including the pelvis, thigh, shank, and foot, were also measured during the experiment. Wearable motion analysis systems (Ultium EMG and Research Pro IMU, Noraxon USA, Inc., Scottsdale, AZ, USA) were used to collect sEMG signals and acceleration data synchronously at a sampling rate of 2000 Hz. A sixteen-camera optical motion capturing system (Vicon Bonita, Vicon Motion System, Ltd., Oxford, UK) was used as a reference system for identifying the locomotion modes. The participants also wore a retroreflective marker set of Plug-in-Gait (PiG) (Figure 1). The marker trajectories were recorded at a sampling rate of 100 Hz. Noraxon MyoSync and Vicon Locker were used to synchronize the instruments and ensure the alignment of the time frames.

### 2.2. Experimental Protocol

**Figure 2:**
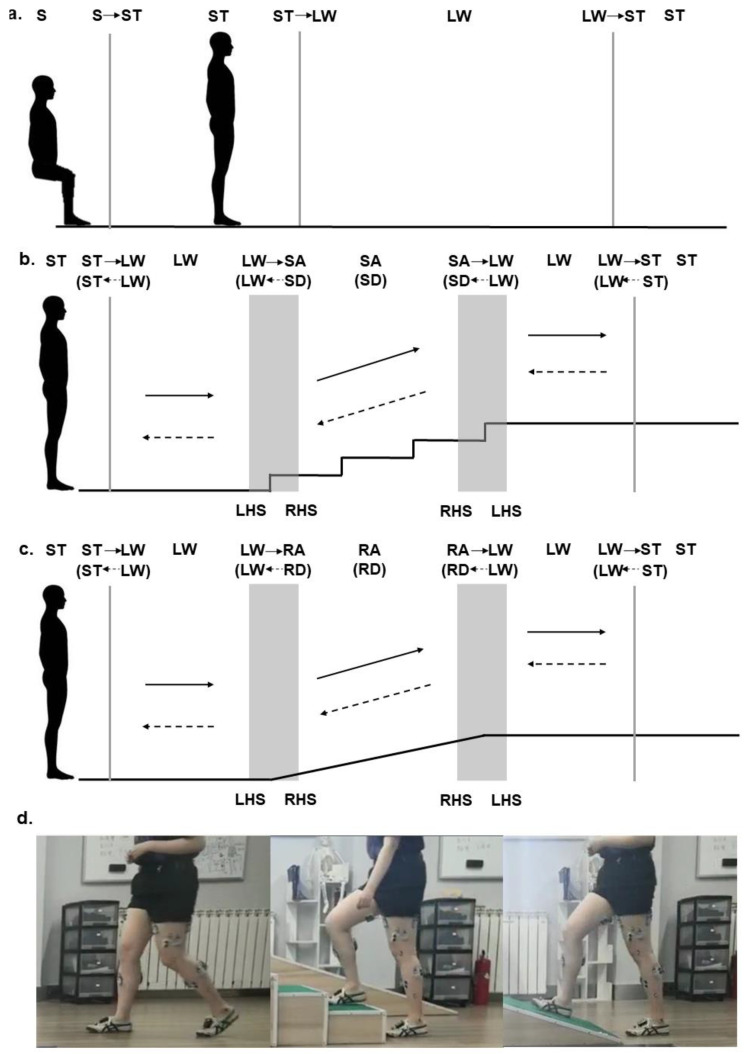
Schematic illustration of the experimental protocol. (**a**) Sit (S)–stand (ST)–level walk (LW)–stand (ST); (**b**) stand (ST)–level walk (LW)–stair ascent (SA)/stair descent (SD)–level walk (LW)–stand (ST); (**c**) stand (ST)–level walk (LW)–ramp ascent (RA)/ramp descent (RD)–level walk (LW)–stand (ST); (**d**) photos of one participant who performed the locomotion transition with her right leg.

Before the experiment, the participants performed a static calibration trial in which they stood straight and still. Seven locomotion activities, including sit (S), stand (ST), level walk (LW), stair ascent (SA), stair descent (SD), ramp ascent (RA) and ramp descent (RD), were studied in the experiment. Each participant was required to complete the following tasks based on daily locomotion activities where different locomotion modes are usually combined (Figure 2): (1) S–ST–LW–ST; (2) ST–LW–SA–LW–ST; (3) ST–LW–SD–LW–ST; (4) ST–LW–RA–LW–ST; (5) ST–LW–RD–LW–ST. The trials were designed based on common daily locomotion activities during daily life. The participants completed 30 trials at comfortable speeds for every task. They ambulated over a 4-step staircase for SA and SD and walked over a ramp with an angle of 30°. The participants were required to initiate and terminate each locomotion activity with the right leg.

### 2.3. Data Processing

Raw sEMG signals were preprocessed using a fourth-order zero-lag Butterworth band-pass filter (30–300 Hz) and a sixth-order zero-lag Butterworth low-pass filter (25 Hz) was used for linear acceleration data. The preprocessed sEMG and acceleration data were then segmented for the different locomotion modes based on the gait cycles. One gait cycle was defined as two consecutive heel strikes (HSs) of the ipsilateral limb. The HS event was detected based on Zeni’s algorithm [17] using trajectory markers of the heel, toe, and pelvis. We defined that a locomotion mode started with foot heel strikes on the terrain and ended when the last HS occurred on the same terrain. If the participant switched locomotion modes during a gait cycle, the gait cycle was regarded as a gait transition, which could be defined using the hip flexion angular velocity obtained from the PiG model. 

The root mean square (RMS) of the sEMG and linear acceleration data was calculated with the use of the sliding window analysis method [18]. The RMS features were then normalized with the minimum and the maximum values in all activities and combined to form a vector as follows:

(1) $$X_{i}=\left\{T_{i\_e1},\ldots ,T_{i\_em},\ldots ,T_{i\_e14},T_{i\_a1},\ldots ,T_{i\_an},\ldots ,T_{i\_a21}\right\}$$

where i represents the ith sample instant, m represents the channel number of sEMG, and *n* corresponds with the 7 channels of the 3-axis linear acceleration data. The $X_{i}$ contained 35 feature samples in total. 

Four machine learning methods were applied in this study to develop locomotion recognition models: support vector machine (SVM), k-nearest neighbor (KNN), artificial neural network (ANN), and linear discriminant analysis (LDA). SVM is defined by a separating hyperplane that maximizes the interval between each class and enables the mapping of features to a high-dimensional feature space through the kernel function to achieve a nonlinear classification. KNN is another common biological signal classifier based on the traditional machine learning algorithm [13] in which features are classified by measuring the Euclidean distance between the different feature vectors. LDA is considered a very simple but effective method and the ANN model enables the description of nonlinear class boundaries between classes [19]. A five-fold cross-validation was used to train our models and the model performance was then evaluated with the use of a separate test dataset. The model parameters used for each machine learning method are shown in Table 1.

**Figure 3:**
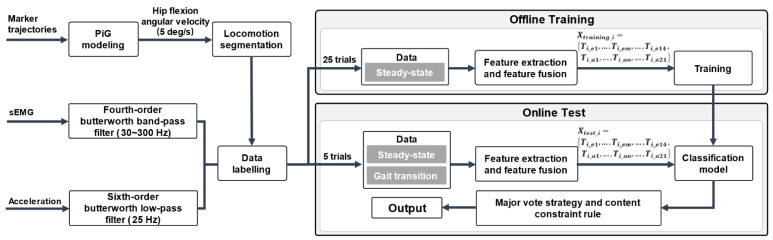
Flowchart of the offline training and online test based on SVM, KNN, LDA, and ANN, respectively.

Figure 3 illustrated the construction, training, and testing of the locomotion recognition models. Thirty trial data of each task were randomly divided into six sets. Five sets were used to train the classifier whereas the other set was used for evaluating the offline and online classification performance. In the online recognition test, a fixed-length window slides along the sEMG and linear acceleration data with a step of one sample, the RMS features are calculated, and online recognition results can be obtained for each sample instant. A major vote strategy and a content constraint rule were used to improve the stability and accuracy of the online performance. The classifier output did not update until at least 100 previous overlapping windows agreed upon a locomotion mode. It needs to be noted that the features were updated when one new data sample fed into the classifier so that the classification model had a common 50 ms delay for locomotion recognition. Moreover, the classification output only updated when the transition of the locomotion modes fitted the transition logic rules, as shown in Table 2. We stipulated that the S mode could only be converted to the ST, the ST could only be transited to the LW, the LW could be converted to any locomotion mode except the S, and the locomotion activities on stairs and ramps could only be converted to the LW. The recognition result of the classifier would then be the output if it met the abovementioned major vote strategy and content constraint rule. The above procedure was carried out using MATLAB software (The Mathworks, Natick, MA, USA).

### 2.4. Data Analysis

The classification accuracy (CA), specificity (SP), and sensitivity (SE) were used for the evaluation of the online recognition performance of the steady-state locomotion, shown in Equation (2):

$$CA=\frac{N_{true}}{N_{all}}\times 100\%$$

$$SE_{i}=\frac{TP}{TP+FN}\times 100\%\;\bar{SE}=\frac{\sum_{i=1}^{K}SE_{i}}{K}\left(i=1,\;\cdots ,7\right)\;$$

(2) $$SP_{i}=\frac{TN}{TN+FP}\times 100\%\;\bar{SP}=\frac{\sum_{i=1}^{K}SP_{i}}{K}\left(i=1,\;\cdots ,7\right)$$

where $N_{true}$ is the number of correctly classified samples, $N_{all}$ is the total number of test samples, and i represents the seven locomotion activities. $\bar{SE}$ and $\bar{SP}$ are the mean sensitivity and specificity of the locomotion recognition for all activities, respectively. 

The predictive accuracy (PA) and response time (RT) were calculated in the online performance validation, as shown in Equation (3):

(3) $$PA=\;\frac{N_{pred\_tran}}{N_{all\_tran}}\times 100\%\;RT=T_{actual}-T_{pred}$$

where $N_{pred\_trans}$ is the number of correctly predicted samples of the next locomotion activity in the gait transition states and $N_{all\_trans}$ is the total sample number of transitions in the trials. $T_{actual}$ is the actual start time of the steady-state locomotion mode and $T_{pred}$ is the moment when the mode is correctly predicted by the models. The negative value of RT illustrates that the model could predict the steady-state locomotion mode ahead of when the mode actually occurred.

In addition, an analysis of variance (ANOVA) was used to investigate the impact of the different classification methods on the locomotion recognition performance and then the difference between the four classifiers was compared by a post-hoc *t*-test. The *t*-test was used to investigate the impact of different data types on the locomotion recognition and prediction performance. The statistical significance was set as *p* < 0.05.

## 3. Results

**Figure 4:**
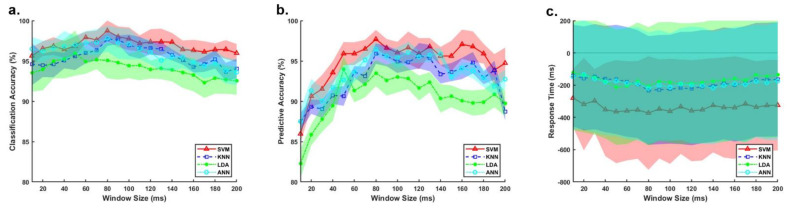
Effects of sliding window sizes and classifiers on: (**a**) the classification accuracy, (**b**) the predictive accuracy, and (**c**) the response time. The results were averaged over 10 participants. The shadows represent the +/– standard deviation (SD).

As shown in Figure 4, the CA and PA increased and RT decreased with an increasing sliding window size until a threshold of approximately 140 ms was reached for all four classifiers. The window size had a greater impact on the prediction performance (varying from 85% to 96%) compared with the recognition of the locomotion modes (from 91% to 97%). All four classifiers performed a high variability in the RT in Figure 4; however, their performance still varied based on the window size. To achieve an optimal online performance, a sliding window size of 80ms was selected for SVM, KNN, and ANN and set as 50ms for LDA for the following investigation. 

The performance of all four classifiers is detailed in Table 3. The results of the one-way ANOVA showed that the classification method had a significant effect on the CA (*p* = 0.0097) in the steady-state locomotion recognition. All four classification methods achieved a high recognition accuracy (CA > 96.4%, SE > 93.6%, SP > 99.4%) and SVM performed the best (CA = 98.78 ± 1.04%, SE = 98.46 ± 0.49%, SP = 99.76 ± 0.04%). It should be noted that only steady-state data were used for the training model and unused transition data could be correctly identified as the next locomotion mode. The methods had a good online locomotion prediction performance during the gait transitions (PA > 93%, RT < −213 ms). However, the prediction performance was significantly different when different models were applied (PA: *p* = 0.0023; RT: *p* = 0.0046). SVM reached the highest PA of 97.69 ± 0.85% in the four classifiers and its RT value (−372.63 ms) was significantly smaller than the other three classifiers (KNN: *p* = 0.0085, LDA: *p* < 0.001, ANN: *p* < 0.001). Overall, SVM had the best performance in the locomotion recognition and prediction. 

**Figure 5:**
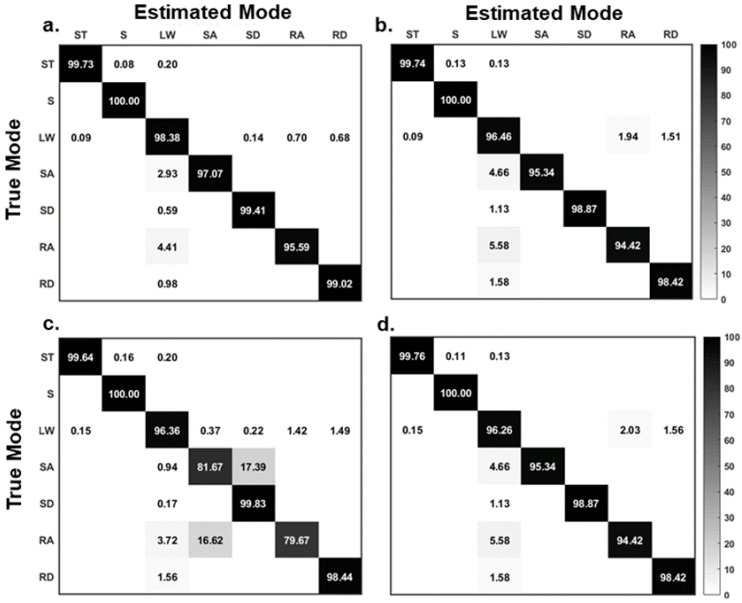
Confusion matrix for the steady-state locomotion recognition of seven locomotion modes for the four classifiers: (**a**) SVM; (**b**) KNN; (**c**) LDA; (**d**) ANN. The results in the confusion matrices were averaged over ten participants. The ST, S, LW, SA, SD, RA, and RD denote standing, sitting, level walking, stair ascent, stair descent, ramp ascent, and ramp descent, respectively.

The confusion matrix for the steady-state locomotion recognition of seven locomotion modes for the four classification methods is shown in Figure 5. We observed that SVM had the highest classification accuracy of the seven locomotion modes (95–100%) followed by KNN (94–100%) and ANN (94–100%). LDA had a large difference in the recognition performance for different locomotion modes (79–100%). The S mode (sitting) was the most distinguishable from other modes. The classifiers performed significantly worse for identifying the RA and SA compared with the others. Most errors in the RA and SA recognition were misclassified as the LW mode (Figure 5).

**Figure 6:**
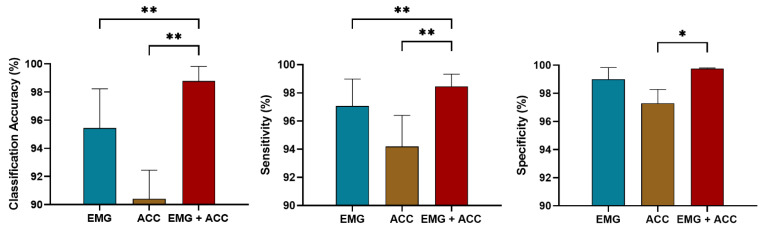
Comparison of the steady-state locomotion recognition performance using sEMG, linear acceleration, and data fusion. * and ** demonstrate a statistically significant difference (*t*-test, *: *p* < 0:05, **: *p* < 0:001).

**Figure 7:**
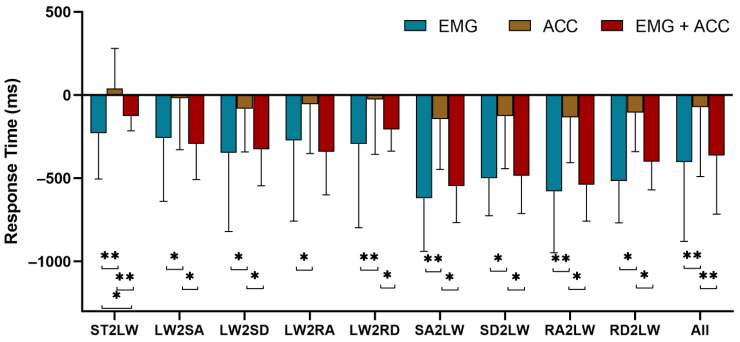
Comparison of the prediction performance using sEMG, acceleration, and sensor fusion signals. ST, S, LW, SA, SD, RA, and RD denote standing, sitting, level walking, stair ascent, stair descent, ramp ascent, and ramp descent, respectively. * and ** demonstrate a statistically significant difference (*t*-test, *: *p* < 0:05, **: *p* < 0:001).

We also compared the online performance of the SVM model with the use of only sEMG, the acceleration and fusion of sEMG, and linear acceleration data. Figure 6 shows that the data fusion method significantly enhanced the classification accuracy (*p* < 0.001) and sensitivity (*p* < 0.001) for the steady-state locomotion recognition compared with that using one type of data alone. SEMG provided essential information for the prediction of locomotion where the performance using the sEMG signals was significantly better than with the use of the linear acceleration data alone (*p* < 0.001), as shown in Figure 7. SVM had a better prediction performance in the gait transitions from other locomotion modes (i.e., SA, SD, RA, RD) to the LW compared with that from the LW to other modes. The combination of sEMG and the acceleration data relatively reduced the response time but no significant statistical difference in most transition modes was observed, as shown in Figure 7. However, the data fusion significantly improved the recognition performance.

## 4. Discussion

SEMG reflects the physiological properties of neuromuscular control in locomotion. In this study, we hypothesized that the use of EMG could enhance the steady-state locomotion prediction during gait transitions and investigated the feasibility of locomotion prediction based on a fusion of sEMG and inertial data. The results demonstrated that sEMG provided essential information for the recognition of locomotion mode prior to initiation.

The role of sEMG fused with inertial data for enhanced locomotion recognition and prediction was firstly investigated in this study. Gao et al. [20] integrated sEMG signals with a ground reaction force in a locomotion recognition model. The method effectively improved the recognition accuracy (96.8%) compared with the model based on only mechanical data (80.96%). The study from Ai et al. [21] showed that a model based on the data fusion of sEMG and acceleration achieved over a 5% higher accuracy in the recognition of five daily activities than that using acceleration data alone. Spanias et al. [22] demonstrated that the combination of EMG and kinematic information enabled a significant reduction of the online error rate for the locomotion mode recognition from 14.1% (mechanical sensor data only) to 7.9% (fusion data). Consistent with previous studies [20,21,22,23,24,25,26,27,28], the results showed that the fusion of EMG signals improved the recognition accuracy of the steady-state locomotion activities from 90% (using acceleration data only) to 98% (using data fusion), which effectively supports the important role of sEMG in locomotion recognition. However, the early activation property of EMG is often overlooked. These studies usually regarded the locomotion transition as an independent state that could be recognized in order to detect the wearer’s intention for the next locomotion. We used the data from the steady-state locomotion to train the models, which could accurately predict the next locomotion activity in advance during the gait transition. This finding supported that sEMG could contribute significantly to locomotion prediction as muscle neural activation occurs prior to the actual movement. 

Regarding locomotion recognition based on fused data, SVM performed the best on the classification and prediction in the four classifiers. Huang et al. [13] fused EMG and GRF to identify six locomotion activities and the results showed that SVM had a higher accuracy than LDA. Li et al. [28] developed a locomotion classification system for lower limb hemiparetic patients. The empirical results demonstrated that SVM (95.2%) produced a better classification accuracy than KNN (89.2%). The outperformance of SVM on locomotion recognition was also pointed out by Zhou et al. [29] and SVM had the highest accuracy (94.29%) compared with KNN and ensemble learning algorithms. The results of our study were consistent with previous studies. As three traditional machine learning methods were utilized, SVM and KNN performed significantly better than LDA in steady-state locomotion recognition and SVM could predict the next steady-state locomotion mode 140–158 ms earlier than the other two models. We additionally employed a neural network approach, which has been suggested to greatly improve the nonlinearity, solving complex problem ability, and accuracy for locomotion recognition [30]. However, ANN had a similar recognition performance to those of SVM and KNN and did not show advantages in the locomotion prediction. The results suggested that neural network methods are not always the most effective for locomotion recognition and prediction based on fused data with limited samples. Traditional machine learning methods such as SVM can achieve an optimal recognition performance.

The results showed that the online performance of the models based on four different machine learning methods was varied, corresponding with the selection of the window size. The trade-off between the PA and RT needs to be considered. A value of 80 ms was selected for SVM, KNN, and ANN whereas the window size was set to 50 ms for LDA in order to achieve an optimal recognition performance. The window size was significantly shorter than those used in previous studies where a data length of 150–300 ms was usually selected [21,27,29,31,32]. These studies chose a long sliding window for achieving a high recognition accuracy but sacrificed the response time. Our study proposed a major vote strategy and a content constraint rule that significantly increased the online performance of the locomotion recognition. The classifiers, especially SVM, obtained a high predictive accuracy (97.69 ± 0.85%) and correct prediction ahead of the next locomotion (−372.63 ± 352.91 ms) with a significantly shorter window size. 

To ensure smooth locomotion transitions of the exoskeleton control model, locomotion recognition models in most studies need to predict the next locomotion mode before the ‘critical moment’ of the gait transition such as the toe off the ground moment during the gait transition from level walking to stair descent [13,33]. A model with the use of the principle of maximum entropy and prior probability based on the fusion of sEMG signals and ground reaction forces was built for the recognition of five locomotion tasks and it could predict the locomotion transitions 410–620 ms in advance [33]. Zhang et al. used a similar approach to predict locomotion transitions 100–300 ms ahead [34]. These studies mainly focused on locomotion prediction and its applications in prosthetic control for amputees where the gait transitions were recognized as independent states based on the next locomotion activity predicted. In our study, we aimed to test the hypothesis that the recognition method enabled the prediction of the motion intention before steady-state locomotion activities with a high accuracy when not using the training data from the gait transitions. We observed that the prediction of locomotion modes varied corresponding with different gait transitions by taking advantage of the neural properties of sEMG that muscles activate before limb movement. The SVM model could predict the LW before the heel off in the transition of ST to LW and before the toe off when moving from stairs and/or ramps to level walking and could recognize the next locomotion mode in the middle swing in the transitions from the LW to the RA or SA. The results verified the effectiveness of sEMG signals for locomotion prediction. 

It should be noted that a few limitations exist in this study. Only the RMS features of sEMG and acceleration data were used in the classification models. The results from [8] showed that the Wilson amplitude (WAMP), mean of amplitude (MA), energy of wavelet coefficient (EWT), and energy of wavelet packet coefficient (EWP) features are distinguished in locomotion activities. A feature fusion method will be considered in future work to further improve the prediction of locomotion activities. Secondly, data from the gait transitions were not used for the model training. The gait transition provided additional information on the locomotion prediction. Due to high variance in muscle activations and limb movements during the gait transitions, the recognition accuracy of the gait transitions was significantly lower than the steady-state locomotion modes [35]. Our locomotion recognition model enabled the prediction of locomotion with a high accuracy (97.69 ± 0.85%) whilst not using training data from gait transitions, which proved the role of sEMG in the locomotion prediction. In following works, we will consider identifying related research on the motion intention prediction of the gait transition. As we focused on discussing the role of sEMG in locomotion recognition and prediction in the current study, the four most commonly used classifiers were selected. The employed classification methods may ignore temporal dynamics, which could be considered to further enhance the classification performance and reduce the time delay. 

## 5. Conclusions

In this paper, we investigated the role of sEMG in locomotion recognition and prediction based on data fusion with acceleration data. Four classifiers were developed and SVM, KNN, ANN, and LDA methods were used. Their performance in steady-state locomotion recognition was compared and SVM achieved the best recognition accuracy. The effects of the window size and data type were further investigated. The results showed that the addition of EMG signals not only improved the online recognition accuracy of the steady-state locomotion activities but also enabled the model built based on the steady-state data to predict the next locomotion activity during gait transitions. Therefore, the neurological properties of EMG should be considered when developing classifiers for locomotion recognition or prediction, which may help to improve the real-time recognition performance and reduce the computational burden. This work has the potential to develop a real-time human–machine interactive control for a smooth gait transition.

## Figures and Tables

**Table 1:** The settings of the parameters in the four classifiers.

Classifiers	Classifier Parameter Settings
**SVM**	The Radial Basis Function (RBF) kernel method and C-SVM were used to build a ‘one-to-one’ multiclassification model; the optimal penalty factor c and kernel function parameter g were obtained using a five-fold cross-validation.
**KNN**	The K value was obtained using a five-fold cross-validation.
**LDA**	The Gauss kernel function was used as the kernel function.
**ANN**	A two-layer network was constructed with 30 hidden nodes and a selected learning rate of 0.01 and optimized with a gradient descent algorithm.

**Table 2:** A set of logic rules for the locomotion transitions. The 0 and 1 values represent whether the specific gait transition was allowed.

		Current Mode						
		ST	S	LW	SA	SD	RA	RD
**Previous Mode**	ST	1	0	1	0	0	0	0
	S	1	1	0	0	0	0	0
	LW	1	0	1	1	1	1	1
	SA	0	0	1	1	0	0	0
	SD	0	0	1	0	1	0	0
	RA	0	0	1	0	0	1	0
	RD	0	0	1	0	0	0	1

**Table 3:** Performance comparison of the four classifiers.

	SVM	KNN	LDA	ANN	*p*-Value
**CA (%)**	98.78 ± 1.04	97.84 ± 1.24	96.43 ± 1.57	97.74 ± 1.04	***0.0097***
**SE (%)**	98.46 ± 0.87	97.61 ± 1.16	93.66 ± 1.43	97.58 ± 1.09	0.0963
**SP (%)**	99.76 ± 0.04	99.62 ± 0.09	99.43 ± 0.21	99.60 ± 0.17	0.2691
**PA (%)**	97.69 ± 0.85	95.91 ± 1.27	93.99 ± 1.44	96.49 ± 0.96	***0.0023***
**RT (ms)**	−372.63 ± 352.91	−232.64 ± 325.60	−213.37 ± 319.81	−218.97 ± 318.13	***0.0046***

Significant differences (*p* < 0.05) for the one-way ANOVA are indicated in bold and italics.

## Data Availability

Not applicable.
